# HIVIntact: a python-based tool for HIV-1 genome intactness inference

**DOI:** 10.1186/s12977-021-00561-5

**Published:** 2021-06-27

**Authors:** Imogen A. Wright, Michael J. Bale, Wei Shao, Wei-Shau Hu, John M. Coffin, Gert U. Van Zyl, Mary F. Kearney

**Affiliations:** 1 Division of Medical Virology, University of Stellenbosch, Tygerberg Hospital, Cape Town, South Africa; 2 HIV Dynamics and Replication Program, CCR, NCI-Frederick, Frederick, MD USA; 3 Weill Cornell Medical College, NY New York, USA; 4 Advanced Biomedical Computing Center, Leidos Biomedical Research, Inc, Frederick National Laboratory for Cancer Research, Frederick, MD USA; 5 Department of Molecular Biology and Microbiology, Tufts University, Boston, MA USA

## Abstract

The characterisation of the HIV-1 reservoir, which consists of replication-competent integrated proviruses that persist on antiretroviral therapy (ART), is made difficult by the rarity of intact proviruses relative to those that are defective. While the only conclusive test for the replication-competence of HIV-1 proviruses is carried out in cell culture, genetic characterization of genomes by near full-length (NFL) PCR and sequencing can be used to determine whether particular proviruses have insertions, deletions, or substitutions that render them defective. Proviruses that are not excluded by having such defects can be classified as genetically intact and, possibly, replication competent. Identifying and quantifying proviruses that are potentially replication-competent is important for the development of strategies towards a functional cure. However, to date, there are no programs that can be incorporated into deep-sequencing pipelines for the automated characterization and annotation of HIV genomes. Existing programs that perform this work require manual intervention, cannot be widely installed, and do not have easily adjustable settings. Here, we present HIVIntact, a python-based software tool that characterises genomic defects in NFL HIV-1 sequences, allowing putative intact genomes to be identified in-silico. Unlike other applications that assess the genetic intactness of HIV genomes, this tool can be incorporated into existing sequence-analysis pipelines and applied to large next-generation sequencing datasets.

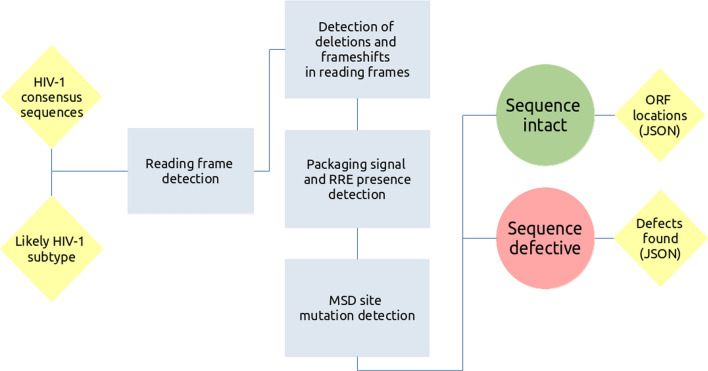

## Introduction: the need for a stand-alone HIV intactness tool that can be integrated into existing pipelines

HIV-1 replication is an error-prone process that often results in the stable integration of aberrant proviruses into the host genome [1]. To date, few consequences of integrated, defective HIV-1 genomes have been described [2]. However, integrated intact genomes are the source of high levels of viremia prior to ART initiation, persistent low-level viremia during ART, and rebound viremia when ART is interrupted. These proviruses are referred to as the HIV-1 reservoir and are the target of potential curative strategies. Analyses of near full length (NFL) proviral genomes on ART reveal that 95–99% contain some manner of defect—usually APOBEC3G-mediated hypermutation or large internal deletions up to 8.5 kb [3–6]. APOBEC-medicated hypermutation typically results in premature STOP codons in high tryptophan regions, as well as numerous missense mutations. Major defects such as hypermutation and large internal deletions are easily identifiable, but minor defects, such as packaging signal deletions, mutations in the major splice-donor site (MSD), or mutations in the rev-response element (RRE) are often observed, but less obvious. Since the vast majority of infected cells on ART contain proviruses with some combination of these lethal defects, interrogation of the HIV-1 reservoir is challenging: one must not only identify infected cells in a background of uninfected cells, but also identify those that contain intact, replication-competent proviruses.

One approach to characterising the HIV-1 reservoir without having to find infected cells is to measure and sequence residual viremia in patients on suppressive ART. Residual viremia is defined as low-level viremia below the threshold of commercial ultrasensitive viral load assays. Cells infected with replication-competent proviruses typically do not express viral RNA during treatment, but a small proportion can become activated to produce virus particles [7]. This activation explains the residual release of viruses, not suppressible by ART, which likely results in viral rebound once treatment is interrupted [8]. Although some defective proviruses could be transcribed and packaged as virions and also contribute to residual viremia [9, 10], in rare cases of persistent clonal viremia high enough to analyze, the virus is replication competent [11, 12]. Another approach to characterising the reservoir is to recover infectious virus from T cells collected from donors on ART using the quantitative viral outgrowth assay (qVOA) [13]. Although highly useful, these techniques have their drawbacks. HIV-1 plasma requires high volume samples and QVOA significantly underestimates the proportion of cells that harbor replication-competent proviruses due to inefficient latency-reversal [3, 14].

Recently, Gaebler et al*.* [14] and Bruner et al*.* [3] proposed two quantitative techniques for measuring the HIV-1 reservoir using qPCR (Q4PCR) and ddPCR (IPDA) respectively. Although these techniques can more accurately quantify the HIV-1 reservoir (reviewed in [15]), both are primer/probe-reliant and have mismatches to some donors, as recently described by Kinloch et al*.* [16]. Although NFL proviral sequencing mitigates some of these issues, it includes its own challenges. For one, the high genetic variation of HIV-1 necessitates the design of large primer panels and sometimes even patient-specific primers. Sanger sequencing approaches, due to their reliance on many primers, are ill-suited to accurately characterize proviral sequences [3, 5, 17–19]. The development of next-generation and third-generation sequencing approaches have overcome these challenges by being less reliant on primers. Deep sequencing is also higher throughput and less expensive than Sanger, making it better suited to NFL analyses [6, 17, 18]. Due to these improvements, it is now possible to assess the putative intactness of many proviral genomes in cells collected from donors on or off ART. However, to date, pipelines that assemble the sequencing reads of NFL HIV-1 genomes do not include a component to annotate the genomes or to infer their intactness.

Although no tools exist that can be incorporated into next-generation sequencing pipelines, there are two freely available tools for the independent bioinformatic determination of HIV-1 intactness, a web-based program called HIV-ProSeqIT [20, 21] and an R-based program called HIVSeqinR [22]. Neither of these tools are intended for high throughput analyses of HIV genomes, and neither can be easily adjusted to include or exclude more stringent checks. Here, we present HIVIntact, a command-line program written in python 3.7 that only requires MAFFT [23] and Biopython [24] to perform an intactness check, allowing ease of integration into existing deep sequencing assembly pipelines. Integration into existing pipelines allows proviral annotation and intactness inference to occur in an automated and high throughput manner, making characterization of the HIV-1 reservoir significantly more accessible. The incorporation of HIVIntact into high throughput methods for NFL proviral single-genome sequencing may constitute the most accurate method to date for measuring and characterizing the HIV-1 reservoir on ART since the NFL sequences can be assessed for minor mutations that are not detected by qPCR and ddPCR assays. Furthermore, the PCR products identified as intact by the tool can be tested in vitro for replication-competence by transfection into permissive cell lines.

## Pipeline definition

### Intact open reading frames (ORFs)

HIVIntact is invoked with a single compulsory parameter: the likely subtype of the NFL HIV-1 sequence. Reference sequences for subtypes A, B, C, D, F, G and H are available by default within the pipeline. The subtype parameter is needed to obtain the best possible estimate of the open reading frames (ORFs) and alignment of accessory genes. Each query sequence is checked for its orientation with respect to the chosen reference sequence. The NFL HIV-1 sequence is aligned in the correct orientation and is first checked for the three large ORFs (*gag,* pro-*pol*, and *env*) in the expected locations. If all three ORFs are present (presence being defined as the absence of a premature stop codon), the sequence is considered to have passed this first “intactness” test. The locations of the ORFs are reported in HXB2 coordinates.

Each large ORF in the candidate intact sequence is then checked for large internal deletions. Deletions amounting to up to 30 bases (consecutive or not) are permitted in *gag* and *pol*, while deletions amounting to up to 100 bases are permitted in *env*, in line with recommendations made in Patro et al. [17] and Shao et al. [20]. The discrepancy in allowed deletion size reflects the greater variability of the *env* gene in vivo. If all three ORFs contain deletions of fewer than the indicated number of bases, then the sequence is considered to have passed this phase of the intactness test.

Each large ORF is then checked for indels that introduce frameshift mutations: a combination of insertions and deletions that shifts the frame. The presence of an indel of any length in a large ORF that shifts the frame results in failure of the intactness test.

Finally, the six smaller ORFs (*vif*, *vpr*, *tat*, *rev*, *vpu*, *nef*) are also checked for “completeness”. In the case of *tat* and *rev*, each of the two exons in the ORF are checked independently. Information on ORF completeness, and the presence of indels and frameshifts within the ORF bounds, is reported. However, because it is not yet known what mutations are tolerated in these genes, this check is not considered by default in the inferred proviral intactness (it may be switched on the command line). Future studies are required to determine the effect of mutations and indels in the small ORFs, so that they can be included in inferred intactness estimations. HIVIntact is well-placed to play a role in these studies by reporting potential defects in these ORFs that may or may not contribute to intactness, which may then be tested in vitro.

### Other genomic structure checks

**Fig. 1:**
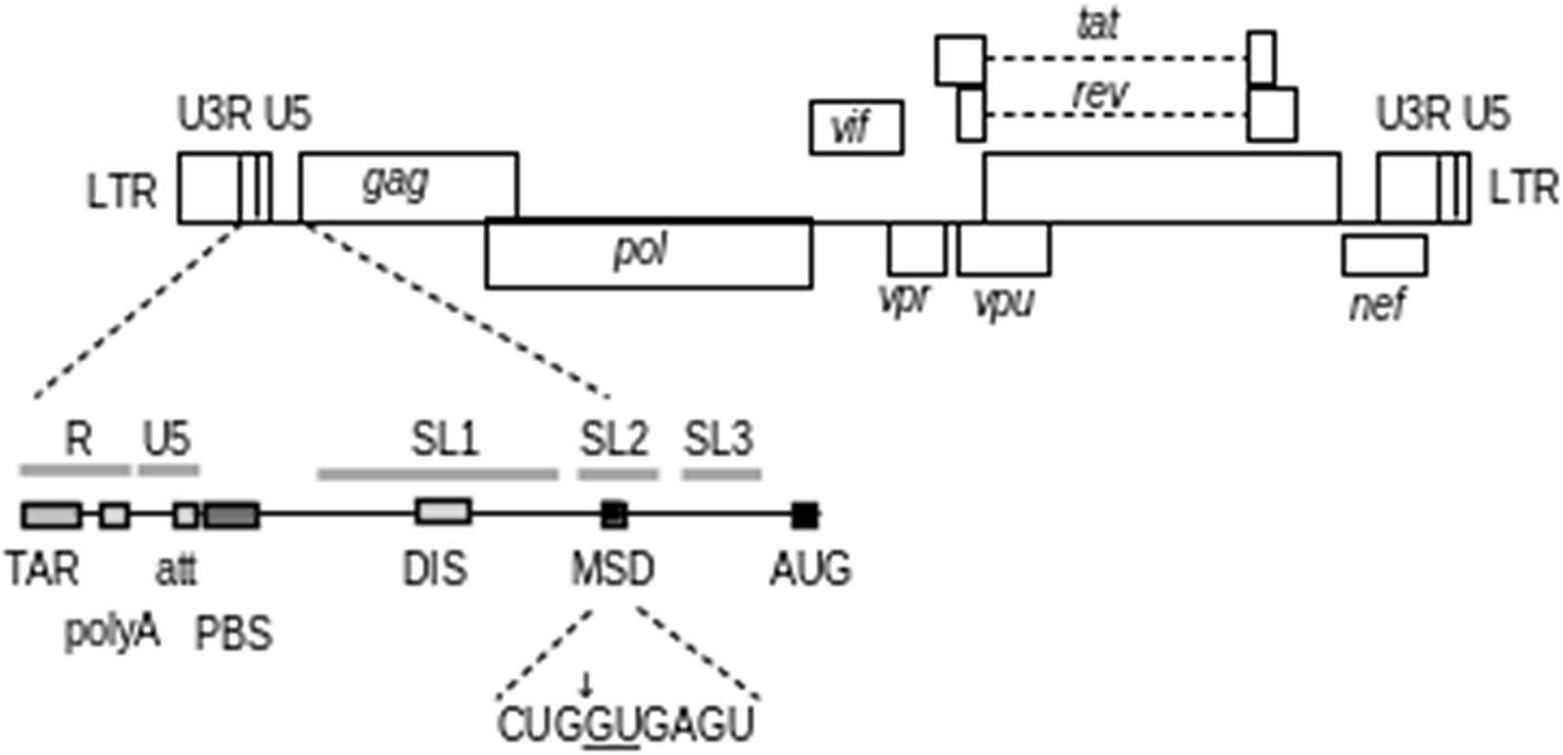
An illustration of the HIV-1 5′ UTR, showing major functional elements, and highlighting the major splice donor site, which must remain unmutated to produce infectious virus

Other functional genomic structures are needed for viral replication in vivo. These include an intact packaging signal (PSI), an unmutated major splice donor (MSD) site (Fig. 1), and intact rev-response element (RRE) [25].

The PSI locus is defined from positions 680–809 in a subtype B reference. A deletion in the PSI as small as 15 nt can render a provirus defective for replication [5]. To err on the side of caution, the defined deletion tolerance in HIVIntact for the PSI is set to 10 nt. Studies are needed to determine the most accurate tolerance for deletions in PSI. We settled on this maximum so as not to omit sequences that should be tested for replication competence in downstream analyses. Users should take note of these estimates in their reports of sequence intactness.

Deletions and mutations in the MSD (located at position 743) have also been demonstrated to render proviruses defective [5, 26]. Because no systematic study has been completed on the effect of all possible MSD mutations, we disallow any mutations in this region. However, both this check and the check for PSI intactness may be disabled by command line arguments.

The RRE locus is defined from positions 7755 to 8020 in a subtype B reference. Because truncations of the first and last 60 nt of the RRE were demonstrated to only reduce replication efficiency by 2.5-fold [27], we chose to allow tolerances in HIVIntact of 39 nt on each end of the RRE and a tolerance of 21 nt insertion/deletion adjacent the ends of the RRE.

More studies are needed to define regions of the RRE that are required for replication competence. Therefore, as with the PSI and MSD checks above, this check may be disabled at the command line by users. Of note, sequences with smaller deletions in domains III–V of the RRE are not included in the pipeline but could render the provirus defective.

### Hypermutation check

An implementation of the HYPERMUT algorithm [28] is included as an additional piece of information. Sequences that fail the Fisher’s exact test for hypermutation are marked as hypermutated in the error output. However, because the intactness of a hypermutated sequence with no premature stop codons in any reading frame cannot be reliably inferred, the hypermutation status of a sequence is not counted towards overall intactness unless the hypermutation introduces a premature stop codon.

## Pipeline implementation

HIVIntact is implemented as a Python 3 script, using Biopython [24] for sequence input and output. The pipeline depends on MAFFT [23] for alignment purposes but is otherwise a standalone tool. The pipeline can be installed globally, as on a high-performance computing cluster, or locally on a personal computer, using a Python package manager.

HIVIntact uses a FASTA file as input. The file should contain one or more assembled NFL HIV provirus sequences. Ideally, these assembled sequences should include coverage of the packaging signal, but a check for its presence is optional and may be switched off on the command line for shorter sequences. The pipeline, once installed, can be called using the proviral intact command, e.g.: proviral intact—subtype B sequences.fasta.

## Pipeline validation

To evaluate the ability of HIVIntact to infer intactness in NFL HIV-1 sequences, we tested all sequences uploaded to the Proviral Sequence Database (PSD) as of 29 September 2020 (https://psd.cancer.gov/intro.php) [20]. The PSD is an existing curated public database of NFL HIV-1 sequences developed and maintained by the National Cancer Institute (NCI). The database contained 4870 sequences at the time of downloading. Of the total sequences, 4143 were unique. The duplicate sequences result from different single-genome sequences obtained from the same donors. Of the unique sequences, 624 were labelled intact in the database and 3519 were labelled defective.

We assessed the full set of 4143 unique sequences with HIVIntact (Table 1). The run completed on a single core of an Intel(R) Core(TM) i7-4770HQ CPU @ 2.20 GHz in 5020 s (1 h, 23 min and 39 s), equivalent to a rate of 1.2 s per sequence per core. This run rate is conducive to automation as part of a larger pipeline.

**Table 1:** A comparison of intactness inference in the NCI PSD [20] with results from HIVIntact

	Inferred intactness in the PSD	1. Reported intactness by HIVIntact (excluding small ORFs)	2. Reported intactness by HIVIntact (including small ORFs)
Intact	624	623	581
Defective	3519	3520	3562
Uniquely intact	3	2	2

The table reports how many sequences were called intact and defective in total in the PSD, as compared against HIVIntact in two modes: (1) assessing only the three major ORFs (*gag*, *pol*, *env*) and (2) including intactness checks for the 6 smaller ORFs (*vif*, *vpr*, *tat*, *rev*, *vpu*, *nef*). The table also reports how many sequences were called intact uniquely by only one tool, indicating a disagreement in intactness inference

## Results excluding small ORFs

We initially ran HIVIntact checking for intactness only in the three major ORFs (*gag*, *pol* and *env*), where defects are well known to render provirus defective. We included checks for defaults in the PSI locus, the RRE locus and the MSD. In this mode, there was very good agreement between our tool and the annotations reported in the NCI PSD.

In total, when considering large ORFs only, five sequences had discordant intactness inference between the PSD and HIVIntact. Three sequences were inferred intact in the NCI PSD but defective by HIVIntact. All three were found to have frameshifts in large ORFs, which is the only default intactness check unique to HIVIntact. Sequence ID MN090882 contained a gag frameshift, while sequences KF526323.1 and MT033880.1 both contained frameshifts in *env.*

Two sequences were inferred defective in the NCI PSD but intact by HIVIntact. Sequence ID MN090886 contained a large, 54-base insertion in the *pol* gene. The PSD considers insertions > 50 bases in *pol* to be defective, while HIVIntact does not currently call sequences with in-frame insertions in the three large ORFs defective. Sequence ID MK114886.1 contains a 10-base deletion in the packaging signal. HIVIntact allows up to 10 base deletions in the packaging signal, while the PSD calls nonintact when the number of deletions is greater than 8.

## Results including small ORFs

We then ran HIVIntact checking for intactness in all 9 ORFS (*gag*, *pol*, *env*, *vif*, *vpr*, *tat*, *rev*, *vpu*, *nef*). In this mode, a further 42 sequences were called nonintact due to defects in one of the 6 small ORFs. Of these errors, 24 were in *vpu*, 9 in *nef*, 6 in *tat, *2 in *vif* and 1 in *vpr.* Further research is needed to quantify which defects in these ORFs genuinely render the virus replication incompetent.

## Pipeline usage

The HIVIntact pipeline and test data may be downloaded from a public GitHub repository (https://github.com/ramics/HIVIntact) under an open-source MIT license. The authors welcome feedback and contributions.

The HIVIntact output includes two FASTA files labeled intact and non-intact. The pipeline also outputs the locations of the ORFs for each sequence despite intactness and a list of defects detected. ORFs and defects are reported in standardised JSON format, allowing bioinformaticians to easily access the results using downstream software applications.

## Data Availability

The HIVIntact pipeline and all test data, including a snapshot of the NCI PSD and a comparison script may be downloaded from a public GitHub repository (https://github.com/ramics/proviral-intactness) under an open-source MIT license, and is available for use. The code is written in Python3 and is platform-independent.
